# Stent-assisted coiling using the Neuroform Atlas stent for treatment of aneurysms that recur after coil embolization

**DOI:** 10.3389/fneur.2022.967942

**Published:** 2022-09-27

**Authors:** Linggen Dong, Jiejun Wang, Xiheng Chen, Longhui Zhang, Zhiqiang Zhao, Qichen Peng, Zeping Jin, Jun Wu, Ming Lv, Peng Liu

**Affiliations:** ^1^Department of Neurointervention, Beijing Neurosurgical Institute, Beijing Tiantan Hospital, Capital Medical University, Beijing, China; ^2^Department of Neurosurgery, Beijing Tiantan Hospital, Capital Medical University, Beijing, China

**Keywords:** Neuroform Atlas stent, recurrent aneurysms, stent-assisted coiling, endovascular treatment, previously coiled

## Abstract

**Objective:**

To evaluate the safety and efficacy of stent-assisted coiling (SAC) using the Neuroform Atlas stent for aneurysms that recur after coil embolization.

**Methods:**

We retrospectively reviewed patients who underwent SAC using the Neuroform Atlas stent to treat aneurysms that recurred after coil embolization from November 2020 to November 2021. Patient and aneurysm characteristics, procedural details, complications, and angiographic and clinical follow-up outcomes were recorded and analyzed.

**Results:**

Eleven patients with 11 recurrent aneurysms were included for analysis. Atlas stent deployment was successful in all cases. Angiography immediately after the SAC procedure and at last follow-up showed complete occlusion in 10 patients (90.9%) and a residual neck in one (9.1%). Mean angiographic and clinical follow-ups were 9.2 and 10 months, respectively. A single procedure-related complication occurred, mildly blurred vision in the left eye, which recovered completely. No permanent morbidity or mortality occurred.

**Conclusion:**

SAC using the Atlas stent to treat aneurysms that recur after coil embolization is safe and effective. Large-scale studies with long-term follow-up are warranted to confirm our results.

## Introduction

Endovascular treatment of intracranial aneurysms is effective; however, recurrence is a known complication that requires re-treatment to reduce the risk of aneurysm growth and hemorrhage (1–3). The reported rates of aneurysm recurrence and re-treatment after coiling are 20% and 10%, respectively (4). Intracranial stents can reduce the incidence of recurrence by preventing coil protrusion into the parent artery, maintaining high coil density within the aneurysm sac, and creating a scaffold for endothelial coverage (5, 6). Several studies have shown that stent-assisted coiling (SAC) is associated with a lower recurrence rate than coiling alone (7).

Since the introduction of the Neuroform stent (Stryker Neurovascular, Fremont, CA, USA) in 2002, intracranial stents have been continually refined. New-generation stents designed with varying structures, lower profile, and improved delivery systems have been introduced to improve aneurysmal occlusion and reduce recurrence (8–10). The self-expanding Neuroform Atlas stent (Stryker Neurovascular) is the successor of the Neuroform stent. This laser-cut stent is made of nitinol and has a mixed open-cell/closed-cell design. Delivery is via microcatheter (0.0165–0.017 inch). The Atlas stent can be used in small distal vessels, which has increased the number of aneurysms amenable to endovascular treatment.

Although efficacy of the Atlas stent has been established in several multicenter studies, to the best of our knowledge, SAC using the Atlas stent for treatment of recurrent aneurysms after coil embolization has not been evaluated (11–13). This study reports our experience.

## Materials and methods

### Patient population

We retrospectively reviewed all patients with intracranial aneurysms treated using the Atlas stent between November 2020 and November 2021 at Beijing Tiantan Hospital. Patients who met the following criteria were included for analysis: (1) age 18 to 80 years; (2) intracranial aneurysm confirmed by digital subtraction angiography (DSA) and previously treated with coiling alone; (3) aneurysm recurrence diagnosed on initial follow-up DSA; (4) re-treatment with SAC using the Atlas stent; and (5) clinical and angiographic follow-up were available after re-treatment. Institutional review board approval was obtained and all patients provided written informed consent.

The following data regarding patient and aneurysm characteristics were recorded: age; sex; hypertension; diabetes mellitus; smoking and alcohol use; symptoms before treatment; history of subarachnoid hemorrhage (SAH); aneurysm location (including bifurcation); irregular aneurysm; aneurysm size; aneurysm neck width; dome/neck ratio; modified Rankin scale (mRS) score before treatment, at discharge, and at follow-up; immediate and follow-up angiographic results; number of stents placed; stent size; procedure-related complications; and interval between initial treatment and re-treatment.

### Endovascular procedure and antiplatelet regimen

Patients with unruptured aneurysms were premedicated with a dual-antiplatelet regimen (clopidogrel 75 mg/d and aspirin 100 mg/d) for at least 5 days. For patients with ruptured aneurysms, we administered loading doses of clopidogrel 300 mg and aspirin 300 mg orally or through a stomach tube 4 h before the procedure. All SAC procedures were performed *via* the femoral approach under general anesthesia and full anticoagulation with heparin (targeted activated clotting time was two to three times above the patient's baseline value). A triaxial guide-catheter system using a 6-Fr Cook (Cook Medical, Bloomington, IN, USA) or 6-Fr Neuron MAX (Penumbra, Alameda, California, USA) long sheath, 5-Fr or 6-Fr Navien (Covidien, Irvine, California, USA) intermediate support catheter, and Excelsior SL-10 or XT-17 microcatheter (Stryker Neurovascular) was used to deploy the stent. Aneurysm morphology and parent arterial structure were assessed using three-dimensional rotational angiography and the proper working projection was selected. An Echelon-10 microcatheter (Medtronic, Dublin, Ireland) was then placed into the aneurysm lumen. An Excelsior SL-10 or XT-17 microcatheter was placed into the parent artery under microguidewire guidance. Aneurysm coiling was performed using the jailing technique. Clopidogrel 75 mg and aspirin 100 mg daily were continued for at least 3 months after the procedure, then aspirin alone for 6 months or life.

### Clinical and angiographic evaluations

Procedure-related complications were categorized as ischemic or hemorrhagic. Ischemic complications were defined as thromboembolic events associated with re-treatment, namely persistent focal neurological deficit, transient ischemic attack, or cerebral infarction. Hemorrhagic complications were defined as visualization of contrast leakage from the aneurysm or ruptured vessel during the procedure or visualization of intracranial hemorrhage on an imaging study performed in the periprocedural period.

Clinical outcome was assessed based on mRS score at last follow-up and was classified as favorable (mRS score 0–2) or poor (mRS score 3–6). Morbidity was defined as any procedure-related neurological deterioration that caused an increase in mRS score.

Angiographic outcomes were evaluated immediately and 6 and 12 months after the procedure using the Raymond–Roy (RR) occlusion classification system: class I, complete occlusion; class II, residual neck; class III, residual aneurysm (14). The outcomes were independently determined by two experienced neuro-interventionalists. Follow-up outcomes were categorized based on comparison with the outcomes immediately after the procedure: (1) improvement, decreased contrast filling in the aneurysm sac; (2) stable, no change in contrast filling; and (3) recurrence, increased contrast filling.

## Results

### Patient and aneurysm characteristics

Eleven patients met inclusion criteria. Median patient age was 49.1 years (range, 31–65) and 8 patients were women. Nine aneurysms presented initially with a rupture. Aneurysm location was anterior communicating artery in 5 patients, posterior communicating artery in 3, pericallosal artery in 2, and superior hypophyseal artery in 1. Mean aneurysm size and neck width was 4.8 ± 2.2 mm (range, 2.7–7.2) and 3.8 ± 1.2 mm (range, 2.5–4.9), respectively. Dome/neck ratio was <2 in all aneurysms and therefore considered wide-necked. Patient and aneurysm characteristics at the time of initial treatment are summarized in Table 1.

**Table 1:** Patient and aneurysm characteristics and procedural details at the time of initial endovascular treatment.

**Patient No**.	**Age (yrs)/Sex**	**Vascular risk factors**	**Initial presentation**	**Aneurysm location**	**Aneurysm type**	**Aneurysm size (mm)**	**Aneurysm neck (mm)**	**Dome/neck ratio**	**RROC scores of the initial treatment**	**Time from initial treatment to Atlas stent placement**
1	65/M	HTN, S,	UIA	PComA	Saccular	7.2	3.4	1.9	1	21 Months
2	57/M	HTN, S, D	RIA	AComA	Saccular	5.3	3.7	0.8	1	54 Months
3	45/M	DM	RIA	Hypophyseal	Saccular	3.8	3.6	0.9	1	117 Months
4	48/F	HTN	RIA	Pericallosal	Saccular	4.3	2.9	0.7	1	11 Months
5	55/F	HTN	UIA	AComA	Saccular	2.7	2.5	0.7	1	14 Months
6	47/F	HTN, S	RIA	Pericallosal	Saccular	3.1	3.0	1.0	1	9 Months
7	48/F	No	RIA	PComA	Saccular	4.3	4.3	0.5	1	20 Months
8	36/F	No	RIA	AComA	Saccular	3.3	3.3	0.7	1	15 Months
9	51/F	HTN	RIA	PComA	Saccular	5.2	4.9	1.1	1	56 Months
10	57/F	HTN	RIA	AComA	Saccular	3.8	3.8	0.7	1	16 Months
11	31/F	No	RIA	AComA	Saccular	3.4	3.4	0.6	1	14 Months

AComA, anterior communicating artery; D, history of drinking alcohol; DM, diabetes mellitus; HTN, hypertension; PComA, posterior communicating artery; RIA, ruptured intracranial aneurysm; S, history of smoking; SAC, stent-assisted coiling; SAH, subarachnoid hemorrhage; UIA, unruptured intracranial aneurysm.

### Technical and angiographic outcomes

Characteristics of the recurrent aneurysms, SAC procedural details, and follow-up outcomes are shown in Table 2. Among the 11 patients with recurrent aneurysms, only one patient (Case 9) presented with a ruptured aneurysm on admission. Atlas stent deployment was successful in all patients. Intraprocedural Dyna computed tomography (Siemens, Munich, and Germany) demonstrated satisfactory vessel wall apposition for all stents. Other than coils, no additional devices such as flow diverters or balloons were used during re-treatment. Immediate postprocedural angiographic showed that complete occlusion (RR class I) was achieved in 10 patients (90.9%), residual neck (RR class II) in 1 patient (9.1%). All patients underwent angiographic follow-up at least once. After a mean of 9.2 months of angiographic follow-up, 10 patients (90.9%) showed complete occlusion (RR class I), and 1 patient showed neck remnant (RR class II).

**Table 2:** Characteristics of recurrent aneurysms, procedural details of stent-assisted coiling, and follow-up outcomes.

**Patient No**.	**Retreatment presentation**	**Retreatment strategy**	**Procedure-related complications**	**Clinical outcome (mRS Score)**		**Angiographic outcome (RROC Score)**		
				**Pre mRS**	**Last F/U mRS**	**Immediate**	**Last F/U**	**F/U Period**
1	UIA	Atlas 3.0 × 21 mm, 10 Coils	No	0	0	1	1	10 Months
2	UIA	Atlas 3.0 × 15 mm, 4 Coils	No	0	0	1	1	8 Months
3	UIA	Atlas 4.5 × 21 mm, 2 Coils	No	0	0	1	1	12 Months
4	UIA	Atlas 3.0 × 21 mm, 5 Coils	No	0	0	1	1	7 Months
5	UIA	Atlas 3.0 × 15 mm, 2 Coils	No	0	0	1	1	8 Months
6	UIA	Atlas 3.0 × 21 mm, 6 Coils	No	0	0	1	1	10 Months
7	UIA	Atlas 4.0 × 15 mm, 2 Coils	No	0	0	1	1	11 Months
8	UIA	Atlas 3.0 × 15 mm, 4 Coils	No	0	0	1	1	7 Months
9	RIA	Atlas 3.0 × 21 mm, 9 Coils	Diplopia after intervention	1	0	2	2	12 Months
10	UIA	Atlas 3.0 × 21 mm, 6 Coils	No	0	0	1	1	9 Months
11	UIA	Atlas 3.0 × 15 mm, 4 Coils	No	0	0	1	1	7 Months

F/U, follow-up; mRS, modified Rankin Scale; RIA, ruptured intracranial aneurysm; RROC, Raymond–Roy occlusion classification; UIA, Unruptured intracranial aneurysm.

### Complications and clinical outcomes

The only complication was minor visual impairment in one patient who developed mildly blurred vision in the left eye after the procedure (mRS score 1). Therefore, clinical outcome was favorable (mRS score 0–2) in all patients at discharge. Mean clinical follow-up was 10 months (range, 6–15). During follow-up, the patient who experienced blurred vision recovered completely (mRS score 0) and the others reported no new neurologic deficits. Follow-up clinical outcome was favorable in all patients without morbidity or mortality.

### Case presentations

#### Case 1

**Figure 1:**
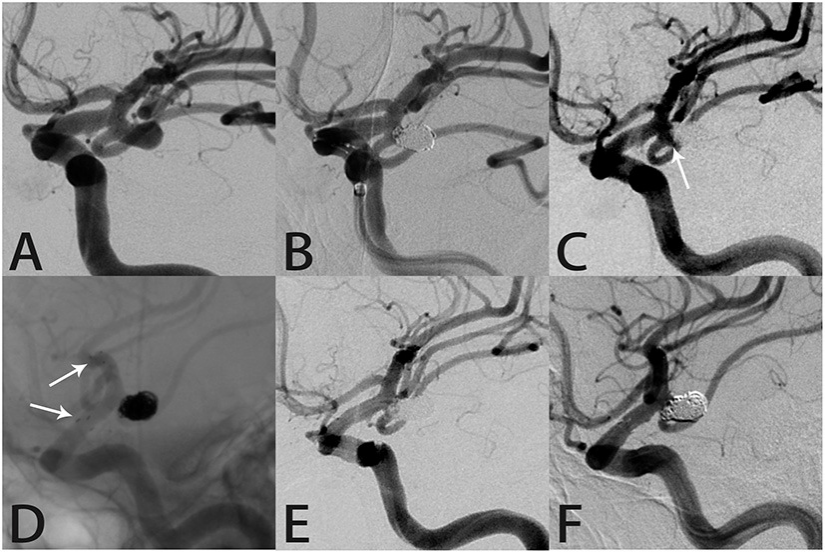
Images from a 65-year-old man with a left posterior communicating artery aneurysm (case 1). **(A)** Preoperative angiography showed a left posterior communicating artery aneurysm. **(B)** The aneurysm was occluded completely after coil embolization. **(C)** Follow-up angiography 21 months after the procedure revealed a mild recurrence in the aneurysm neck (white arrow). **(D)** Angiography during re-treatment showed the deployed Atlas stent (3.0 × 21 mm) covering the aneurysm neck and coils densely packed within the sac (white arrow indicates the end of the stent). **(E)** Angiography immediately after the procedure showed the aneurysm was occluded completely. **(F)** Follow-up angiography 10 months later showed complete aneurysmal occlusion and parent artery patency.

A 65-year-old man presented to an outside hospital with a 1-month history of dizziness and headache. Magnetic resonance angiography showed a left posterior communicating artery aneurysm and he was transferred to our hospital. DSA confirmed the aneurysm (4.8 × 6.7 mm) and RR class I occlusion was achieved with coil embolization (Figures 1A,B). Aneurysm recurrence was diagnosed 21 months later (Figure 1C) and SAC using the Atlas stent (3.0 × 21 mm) was performed without complications. Intraprocedural angiography showed the three radiopaque markers at the proximal and distal ends of the Atlas stent (Figure 1D). Angiography immediately after the procedure revealed RR class I occlusion and dense coil packing within the aneurysm (Figure 1E). Follow-up DSA 10 months later still showed complete aneurysmal occlusion and a patent posterior communicating artery (Figure 1F).

#### Case 2

**Figure 2:**
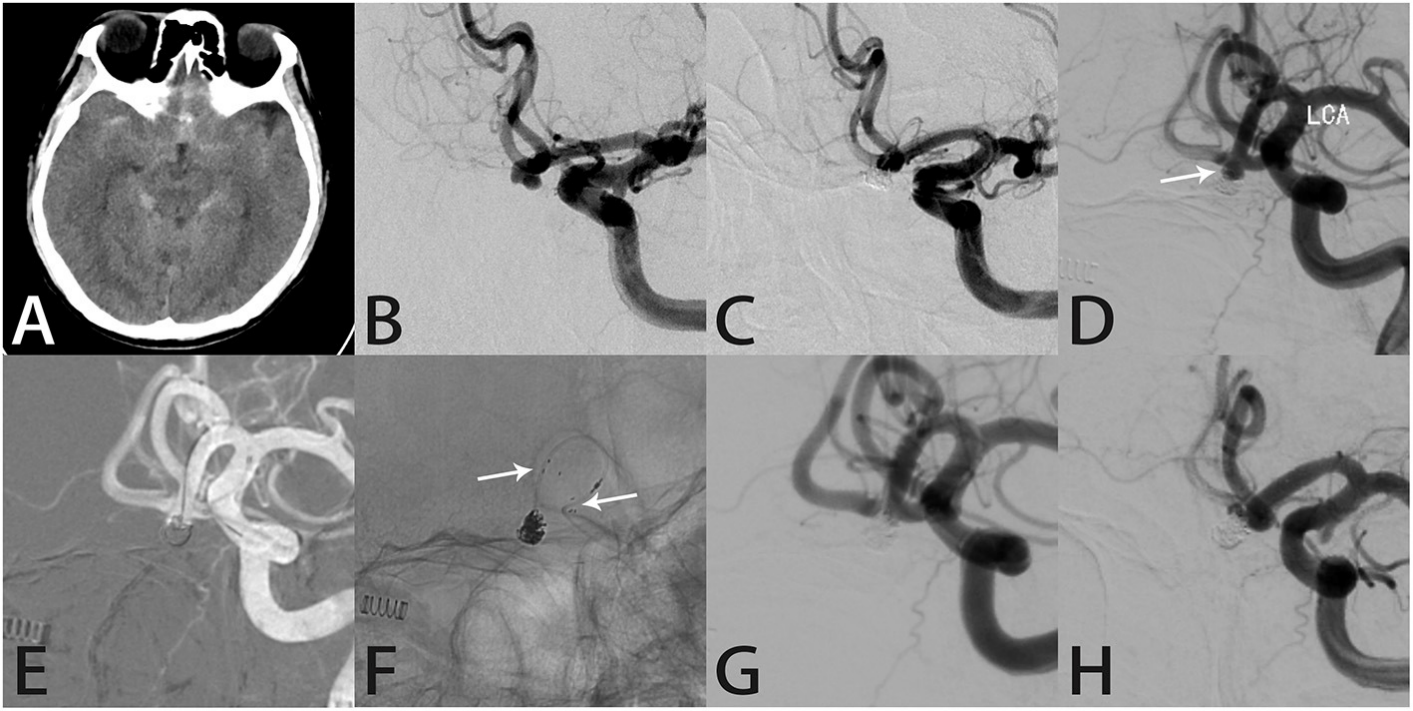
Images from a 57-year-old man with an anterior communicating artery aneurysm (case 2). **(A)** Computed tomography showed subarachnoid hemorrhage in the longitudinal and right Sylvian fissures as well as around the brainstem. **(B)** Preoperative angiography showed an anterior communicating artery aneurysm. **(C)** The aneurysm was occluded completely after coil embolization. **(D)** Follow-up angiography 54 months later showed an obvious recurrence in the aneurysm neck (white arrow). **(E)** During re-treatment, an Echelon 10 microcatheter (Medtronic, Dublin, Ireland) was delivered into the aneurysm sac to place the coils. **(F)** Intraprocedural angiography showed the three radiopaque markers (white arrows) at the proximal and distal ends of the Atlas stent (3.0 × 15 mm) and the coils within the aneurysm sac. **(G)** Angiography immediately after the procedure showed the aneurysm was occluded completely. **(H)** Follow-up angiography 8 months later showed complete aneurysmal occlusion and parent artery patency.

A 57-year-old man was admitted to the hospital with a severe headache. Computed tomography showed SAH in the longitudinal and right Sylvian fissures as well as around the brainstem (Figure 2A). DSA showed a saccular anterior communicating artery aneurysm (Figure 2B). Coil embolization was performed, which achieved RR class I occlusion and relieved the headache (Figure 2C). Follow-up angiography 54 months later showed contrast within the aneurysm neck (Figure 2D). During re-treatment, an Echelon 10 microcatheter (Medtronic, Dublin, Ireland) was delivered into the aneurysm sac to place the coils with stent assistance (Figure 2E). Intraprocedural angiography showed the three radiopaque markers at the proximal and distal ends of the Atlas stent (3.0 × 15 mm) and coils within the sac (Figure 2F). Angiography immediately after embolization showed RR class I occlusion (Figure 2G). The aneurysm remained completely occluded and the parent artery was patent on follow-up angiography 8 months later (Figure 2H).

#### Case 4

**Figure 3:**
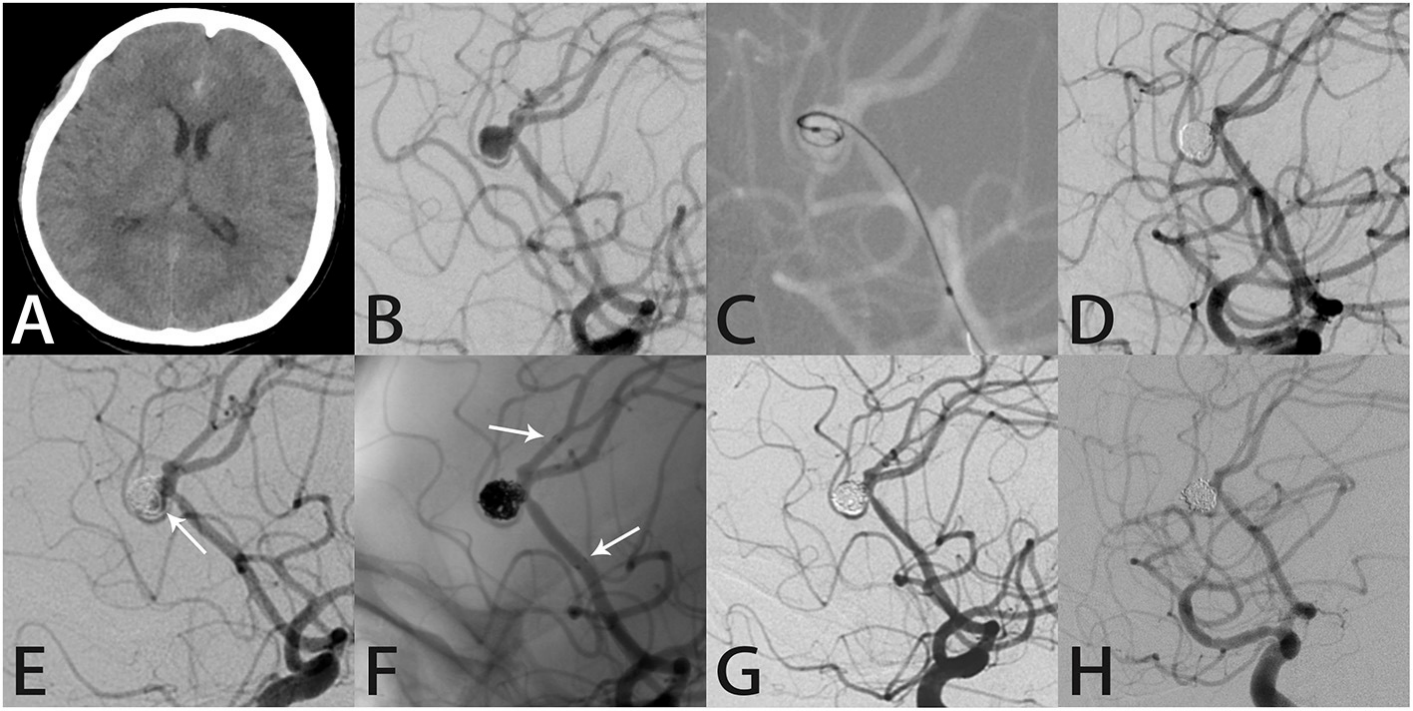
Images from a 48-year-old woman with a right pericallosal aneurysm (case 4). **(A)** Computed tomography showed subarachnoid hemorrhage in the longitudinal fissure. **(B)** Angiography showed a right pericallosal aneurysm. **(C)** During treatment, an Echelon 10 microcatheter (Medtronic, Dublin, Ireland) was delivered into the aneurysm sac to place the coils. **(D)** The aneurysm was occluded completely after coil embolization. **(E)** Follow-up angiography 11 months later showed recurrence in the aneurysm neck (white arrow). **(F)** Intraprocedural angiography showed the deployed Atlas stent (3.0 × 21 mm) covering the aneurysmal neck and coils densely packed within the sac (white arrows indicate the ends of the stent). **(G)** Angiography immediately after the procedure showed the aneurysm was occluded completely. **(H)** Follow-up angiography 7 months later showed complete aneurysmal occlusion with the coils densely packed within the aneurysm.

A 48-year-old woman presented with severe headache. Computed tomography showed SAH in the longitudinal fissure (Figure 3A). DSA showed a right pericallosal aneurysm (Figure 3B). Coil embolization was performed (Figure 3C). RR class I occlusion was shown on angiography performed immediately after treatment (Figure 3D). Aneurysm recurrence was diagnosed 11 months later on follow-up angiography (Figure 3E). Because of the complexity of the aneurysm, we elected to perform SAC using the Atlas stent, which was successful without complications. Intraprocedural angiography showed the three radiopaque markers at the proximal and distal ends of the Atlas stent (3.0 × 21 mm) and coils within the sac (Figure 3F). Post-embolization angiography showed RR class I occlusion (Figure 3G). Seven months later, the aneurysm remained completely occluded and the parent artery was patent (Figure 3H).

#### Case 9

**Figure 4:**
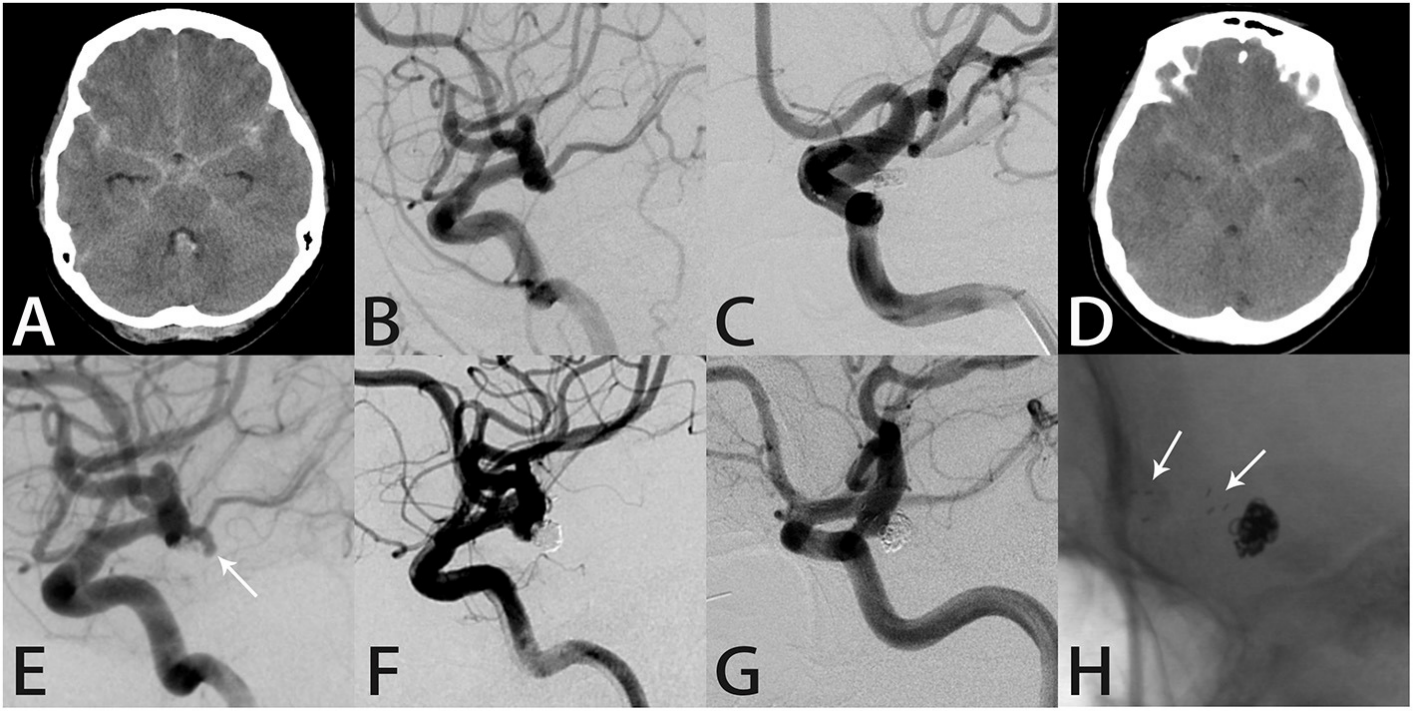
Images from a 51-year-old woman with a left posterior communicating artery aneurysm (case 9). **(A)** Computed tomography showed subarachnoid hemorrhage in the brain basal cistern, lateral fissure cistern, longitudinal fissure cistern, and ambient cistern. **(B)** Angiography showed a left posterior communicating artery aneurysm. **(C)** The aneurysm was occluded completely after coil embolization. **(D)** Computed tomography showed subarachnoid hemorrhage in the lateral fissure cistern and sulcus. **(E)** Angiography showed recurrence of the left posterior communicating artery aneurysm. **(F)** Angiography immediately after re-treatment showed RR class II occlusion. **(G)** Follow-up angiography 12 months later showed the aneurysm remained RR class II occlusion. **(H)** Intraprocedural angiography showed the Atlas stent remained stable and the three radiopaque markers (white arrows) could be seen at the proximal and distal ends of the Atlas stent (3.0 × 21 mm).

A 51-year-old woman suddenly developed a severe headache with nausea and vomiting, followed by loss of consciousness, and was conscious for about 1 h later. Computed tomography performed in our hospital showed SAH in the brain basal cistern, lateral fissure cistern, longitudinal fissure cistern, and ambient cistern (Figure 4A). DSA showed a left posterior communicating artery aneurysm (Figure 4B). We performed coil embolization of the aneurysm, and immediate post-procedural angiographic showed that the aneurysm achieved RR class I occlusion (Figure 4C). The patient recovered well after the procedure, and the headache symptoms recovered at the time of discharge. However, the patient was re-admitted with severe headache 56 months after the initial coil embolization. Computed tomography confirmed that the patient was re-bleeding, and the SAH involved the lateral fissure cistern and sulcus (Figure 4D). The patient had Fisher score of grade 3 and a WFNS score of grade 1. DSA showed recurrence of the left posterior communicating artery aneurysm (Figure 4E). Considering that this patient was re-bleeding and led to recurrence 56 months after the initial coil embolization, after the discussion of several experts in our group, we finally decided to adopt the Atlas stent-assisted coiling treatment strategy. Atlas stents can reduce the incidence of recurrence by preventing coil protrusion into the parent artery, and the low metal coverage rate of the stent may also reduce the incidence of thromboembolic complications. Thus, we performed SAC using the Atlas stent (3.0 × 21 mm), and angiography immediately after re-treatment showed RR class II occlusion (Figure 4F), and the patient developed mildly blurred vision in the left eye after the procedure. Twelve months later, the follow-up angiography showed the aneurysm remained RR class II occlusion and the Atlas stent was stable (Figures 4G,H). Notably, the patient's vision was completely recovered.

## Discussion

The effectiveness of the Atlas stent in assisting coil embolization to achieve aneurysm occlusion with low rates of recurrence and morbidity has made it an appealing adjunct when treating intracranial aneurysms (12, 15, 16). As the incidence of aneurysm recurrence has increased in conjunction with increased use of endovascular treatment, so has the use of SAC with the Atlas stent to treat these recurrences. Currently, few data are available regarding SAC using the Atlas stent for treatment of recurrent aneurysms. Thus, in this study, we describe our experience spanning 1 year at a single center with the safety and efficacy of SAC using the Atlas stent to treat recurrent aneurysms after coil embolization.

Since the publication of the International Subarachnoid Aneurysm Trial, the paradigm for intracranial aneurysm treatment has gradually shifted from microsurgical clipping to endovascular intervention (17). Endovascular coil embolization is now widely used and is well-known for its low morbidity and mortality (18). However, risk of recurrence remains a major concern. A systematic review reported that 10% to 33.6% of all cerebral aneurysms treated with endovascular coil embolization recur and re-treatment rates range between 4.7 and 12.3% (19). In addition, recurrent aneurysms are associated with a higher rate of intracranial hemorrhage. Slob et al. (20) reported a 6.9% hemorrhage rate in patients with incompletely occluded aneurysms after initial coiling who were not re-treated; however, the rate was zero in those who underwent additional coiling. Therefore, re-treatment of recurrent aneurysms appears to reduce the risk of future hemorrhage.

Several endovascular treatment modalities are available to manage recurrent aneurysms, including re-coiling, SAC, and placement of a flow diverter. Previous studies have found that re-embolization using coils alone is associated with a higher rate of recurrence (21, 22). Stent assistance stabilizes the inserted coils, maintains parent artery patency, and provides protection against recurrence (22). Therefore, SAC may be a better alternative. Daou et al. (23) reported an 86.7% complete or near-complete occlusion rate after placement of the Pipeline embolization device (Medtronic, Dublin, and Ireland) in previously coiled recurrent aneurysms, demonstrating the efficacy of flow diversion. However, another study reported high rates of complications (17.2%) and permanent morbidity (6.9%) with this approach (24). Therefore, neurointerventionalists should carefully consider the appropriate treatment modality on an individual basis when managing recurrent aneurysms.

Re-treating recurrent aneurysms is frequently technically challenging. Accurate measurement of the recurrent lumen may be difficult owing to the previously inserted coils (25). Furthermore, if thrombus has formed within the lumen, it may dislodge into the parent artery and cause a serious thromboembolic complication (25). Moreover, many recurrent aneurysms have a wide neck and relatively shallow depth because of coil compaction (22). Therefore, the procedure may require complex manipulations through multiple microcatheters and stent assistance may be necessary to prevent coil protrusion and migration. Our technical success rate for SAC using the Atlas stent was 100%, which is comparable to previously reported rates for treatment of naïve aneurysms (5, 26). The Atlas stent can be successfully used for SAC of previously coiled aneurysms.

Our rates of RR class I and II occlusion immediately after the procedure were 90.9 and 9.1%, respectively, and these rates remained stable at the last follow-up. We attribute the favorable angiographic outcomes to our use of Atlas stent assistance. These rates compare favorably with other multicenter studies that have evaluated Atlas stent performance in SAC of naïve aneurysms (12, 16, 27). The rate of complete occlusion in these studies ranged from 81.3 to 86.7%. In addition, our results are comparable to those achieved in a multicenter study of SAC using the Acclino stent (Acandis GmbH, Pforzheim, Germany) to treat recurrent and residual aneurysms; this study reported a 94.7% complete occlusion rate immediately after the procedure that decreased to 76.9% at last angiographic follow-up (28). Previous studies of SAC using the LVIS Jr (MicroVention, Aliso Viejo, California, USA) and LEO Baby (Balt Extrusion, Montmorency, France) stents have focused on the treatment of naïve aneurysms (8, 29); they have not been examined yet for treatment of recurrent aneurysms.

Procedure-related complications occurred in only one patient (9.1%), a 51-year-old woman with a recurrent left posterior communicating artery aneurysm who developed mildly blurred vision in the left eye. Angiography immediately after re-treatment showed RR class II occlusion. Although her vision completely recovered, follow-up angiography remained RR class II occlusion. We believe her blurred vision may be attributed to oculomotor nerve palsy (ONP). Unilateral ONP occurs in approximately 25% of patients with a posterior communicating artery aneurysm (30) and often occurs at the time of aneurysm rupture. The cause may be rupture-related trauma to the nerve or the presence of localized hematoma and/or subarachnoid blood (31). However, ONP may also be observed in association with unruptured aneurysms because of direct mechanical compression from the aneurysm sac and/or aneurysm pulsatility (32). Considering that this patient's aneurysm was relatively small and unruptured, we presume that the most likely cause was aneurysm pulsatility. A previous study reported that coiling can eliminate aneurysmal pulsations, which allows more complete nerve recovery (31). This patient's vision recovery supports this hypothesis.

Two previous studies of SAC using the Atlas stent for treatment of naïve aneurysms have reported thromboembolic complication rates of 3.8 and 2.3%, respectively, and hemorrhagic complication rates of 0.8 and 0.8%, respectively (33, 34). Reported thromboembolic complication rates in SAC studies using the Neuroform and Enterprise (Codman Neurovascular, Raynham, MA, USA) stents were 8.8 and 8.7%, respectively (35, 36). The Atlas stent appears to be associated with a lower rate of thromboembolic complications than conventional stents. One possible explanation is that its miniaturized design and delivery system reduces exposure of the metal-covered surfaces to blood flow, which reduces thrombus formation.

All patients who initially presented with SAH in our study were treated using coil embolization alone, suggesting that most neurointerventionalists are reluctant to use stent assistance when embolizing acutely ruptured aneurysms. This is understandable because the body is in a hypercoagulable state in the acute stage of SAH and introduction of a stent into the cerebral vasculature may increase the risk of thromboembolism. Furthermore, the antiplatelet therapy that is generally associated with stent placement may increase the risk of re-hemorrhage, especially in patients with an intraventricular catheter. In patients with a ruptured aneurysm, the rate of ventriculostomy-related re-hemorrhage is 3.4 times higher in those who undergo SAC than in those who undergo coiling alone (37). Therefore, stent use should be considered with caution in ruptured aneurysm patients.

Although the results of this study are encouraging, future large-scale studies with long-term follow-up are needed to fully evaluate the efficacy of SAC using the Atlas stent for recurrent aneurysms.

### Limitations

This study has several limitations. Its retrospective single-center design lacked a control group and selection bias may have been introduced. In addition, its sample size was small and the follow-up period was short; therefore, our reported occlusion rates may not accurately reflect the true rates.

## Conclusion

SAC using the Atlas stent to treat aneurysms that recur after coil embolization is safe and effective. However, large-scale studies with long-term follow-up are necessary to validate our results.

## Data availability statement

The raw data supporting the conclusions of this article will be made available by the authors, without undue reservation.

## Ethics statement

This study was reviewed and approved by the Ethics Committee of Beijing Tiantan Hospital. Written informed consent to participate in this study was provided by the patients or their legal guardian/next of kin.

## Author contributions

JW, ML and PL: conception and design. XC, LZ, ZZ, QP, and ZJ: data analysis and interpretation. LD and JW: manuscript writing. The final version was approved by ML on behalf of all authors. All authors contributed to the article and approved the submitted version.

## Funding

This study was supported by the Youth Program of National Natural Science Foundation of China (Grant No. 81901197) and National Key Research and Development Program of the 14th Five-Year Plan (Grant No. 2021YFC2501100).

## Conflict of interest

The authors declare that the research was conducted in the absence of any commercial or financial relationships that could be construed as a potential conflict of interest. The reviewer YW declared a shared parent affiliation with the authors to the handling editor at the time of review.

## Publisher's note

All claims expressed in this article are solely those of the authors and do not necessarily represent those of their affiliated organizations, or those of the publisher, the editors and the reviewers. Any product that may be evaluated in this article, or claim that may be made by its manufacturer, is not guaranteed or endorsed by the publisher.
